# Concordance between late effects reported by physicians and patients in a cohort of long-term Hodgkin lymphoma survivors: an analysis of data from nine consecutive EORTC-LYSA trials

**DOI:** 10.1007/s11764-024-01694-0

**Published:** 2024-10-18

**Authors:** Sidsel J. Juul, Sára Rossetti, Berthe M. P. Aleman, Flora E. van Leeuwen, Marleen A. E. van der Kaaij, Francesco Giusti, Paul Meijnders, John M. M. Raemaekers, Hanneke C. Kluin-Nelemans, Michele Spina, Daphne Krzisch, Camille Bigenwald, Aspasia Stamatoullas, Marc André, Wouter J. Plattel, Martin Hutchings, Maja V. Maraldo

**Affiliations:** 1 Department of Oncology, Copenhagen University Hospital-Rigshospitalet, Blegdamsvej 9, 2100 Copenhagen, Denmark; 2 Department of Haematology, Copenhagen University Hospital-Rigshospitalet, Copenhagen, Denmark; 3 Department of Radiation Oncology, The Netherlands Cancer Institute, Amsterdam, The Netherlands; 4 Department of Psychosocial Research and Epidemiology, The Netherlands Cancer Institute, Amsterdam, The Netherlands; 5 Department of Internal Medicine, Amstelland Hospital, Amstelveen, The Netherlands; 6 EORTC Headquarters, Brussels, Belgium; 7 Department of Radiation Oncology, Iridium Network, University of Antwerp, Antwerp, Belgium; 8 Department of Haematology, Radboud University Medical Center, Nijmegen, The Netherlands; 9 Department of Haematology, University Medical Centre Groningen, University of Groningen, Groningen, The Netherlands; 10 Division of Medical Oncology and Immune-Related Tumors, IRCCS Centro Di Riferimento Oncologico Di Aviano (CRO), IRCCS, Aviano, Italy; 11 AP-HP, Hôpital Saint-Louis, Hemato-Oncologie, DMU DHI, Université de Paris, F-75010 Paris, France; 12 Department of Haematology, Institute Gustave Roussy, Villejuif, France; 13 Department of Haematology, Centre Henri Becquerel, Rouen, France; 14 Department of Haematology, CHU UCL NAMUR, Yvoir, Belgium; 15 Department of Clinical Medicine, University of Copenhagen, Copenhagen, Denmark; 16 Present Address: Sciensano, Brussels, Belgium

**Keywords:** Hodgkin lymphoma, Late effects, Survivorship, Patient-reported outcomes, Concordance

## Abstract

**Purpose:**

Studies looking into the concordance between late effects reported by physicians vs. those reported by Hodgkin lymphoma (HL) survivors are missing.

**Methods:**

A Life Situation Questionnaire focusing on late effects collected data from 1230 HL survivors (median follow-up 14.3 years). Twenty-six disease- and treatment-related late effects from various organ systems were matched with physician-recorded data. The concordance between physicians and survivors was systematically evaluated using percentage agreement and kappa statistics. Potential non-responder biases and associations with patient and disease characteristics were also investigated.

**Results:**

Agreement levels (indicated by kappa statistics) varied from none to moderate agreement, with the highest Kappa values observed for myocardial infarction (kappa = 0.55, 95% CI 0.43–0.66) and pulmonary embolism (kappa = 0.55, 95% CI 0.35–0.75). HL survivors consistently reported a higher prevalence of late effects compared with physicians. Notably, the prevalence of subjective symptoms such as persistent fatigue and xerostomia was repeatedly underreported by physicians. A trend towards higher concordance was observed in survivors with higher clinical stage, higher education level, and treatment initiated at younger ages. Additionally, findings indicated that survivors who did not respond to the questionnaire experienced fewer late effects compared to those who did respond.

**Conclusions:**

Substantial discrepancies were noted in the reported prevalence of late effects between survivors and physicians, especially for outcomes which are not easily quantified.

**Implications for Cancer Survivors:**

It is therefore essential to integrate outcomes reported by both physicians and survivors to achieve a comprehensive assessment of the long-term consequences of HL treatment.

**Supplementary Information:**

The online version contains supplementary material available at 10.1007/s11764-024-01694-0.

## Introduction

Advances in treatment have led to substantial improvements in survival rates for Hodgkin lymphoma (HL) patients [1]. Monitoring of therapy-induced late effects, therefore, becomes increasingly relevant. However, the information available to clinicians and their adult patients regarding adverse effects of cancer treatment is mostly based on reports made by clinicians rather than on reports from the patients themselves [2, 3]. In symptom research (where patient reporting is considered the gold standard for evaluating symptoms), studies have shown that physicians and nurses consistently underestimate symptom frequency or severity when compared with patient ratings [4–6]. As a consequence, underreporting may be significant [2].

A precise description of the prevalence and severity of late effects is essential for an informed evaluation of anticancer treatment [2]. While the concordance between patient reports and medical documentation has been studied extensively for adverse events that occur during treatment [5, 7–9], the concordance between physician and cancer survivor–reported outcomes on long-term late effects remains undocumented.

The updated European Organisation for Research and Treatment of Cancer (EORTC) Lymphoma Group database provides detailed information on late effects from both patients and physicians. This unique dataset creates an unparalleled opportunity to link patient-reported outcome information to physician reports enhancing our understanding of survivorship. In this retrospective study, we aim to investigate the concordance between long-term late effects reported by physicians to those reported by HL survivors. Associations with patient and disease characteristics and potential non-responder biases are also investigated.

## Methods

### Study design and patients

To improve the outcome for HL patients, the EORTC Lymphoma Group has performed clinical trials since 1964, randomizing 6658 patients in its first nine trials (from 1993 onwards, in collaboration with the Lymphoma Study Association (LYSA)). Details of treatment and results of the individual trials (H1-H9) have been documented in separate publications [10–19]. To study the subjective perception of late effects after HL treatment, a cross-sectional study (Life Situation Questionnaire (LSQ)) was conducted between 2009 and 2011. The extensive LSQ was sent to all patients from the nine trials known to be alive and with a registered address. Current addresses were found for more than 3600 surviving patients [20]. Additionally, a medical survey was completed between 2014 and 2019 to collect physician-reported follow-up data on treatment-related toxicity in the same patient cohort. The medical survey was sent to all H1–H9 participating centers where a principal investigator was available (for further information, see supplement S1). In the LSQ, late effects were assessed via questions such as “Do you have a thyroid gland which is working too slowly”? “Do you have high blood pressure” or “Dryness of the mouth (duration at least a year)”? corresponding to “Hypothyroidism,” “Hypertension,” and “Xerostomia (> 1 year)” respectively, reported in the medical survey. Relevant conditions in the LSQ for which a matching symptom in the medical survey could be found were included in the analysis. Information on patient characteristics and treatment exposure was extracted from the EORTC Lymphoma Group database. Information on late effects was derived from the LSQ and the medical survey. In total, 26 conditions from various organ systems were evaluated.

### Statistical analysis

The methods were adopted from Sikorski et al. [8]. Two binary outcome variables were explored. One variable reflected the documentation of a late effect in the LSQ (yes, as indicated by year of diagnosis, vs. no); the other reflected the presence of the same condition in the medical survey (yes vs. no). Survivors who had the specific conditions prior to the onset of HL were excluded from the analyses, and dates in the medical survey were truncated to match the LSQ (dates available for 17 out of 26 conditions). Several methods were applied to assess the concordance between the two variables as no single numerical summary fully describes the agreement or disagreement [8, 21, 22]. First, Kappa statistics were used to quantify the level of agreement based on the classification system proposed by Landis and Koch [8, 23, 24]. The levels of agreement were classified as no agreement (< 0), slight agreement (0–0.20), fair agreement (0.21–0.40), moderate agreement (0.41–0.60), substantial agreement (0.61–0.80), and almost perfect agreement (0.81–1.00) [23, 24]. Second, the percent agreement was computed for each of the 26 conditions. This was done by dividing the number of survivors for whom the condition was either present or absent in both the LSQ and the medical survey by the total number of HL survivors. Consequently, the overall percentage agreement mirrors both the presence and the absence of the condition. Percentages for positive and negative agreement were also computed. As neither the LSQ nor the medical survey could be regarded as the “gold standard,” proportions of agreement were calculated for the average of their positive (Ppos) and negative (Pneg) responses [21, 22]. Thus, Ppos was calculated by dividing the number of survivors for whom a condition was present in both the LSQ and the medical survey by the average number of positive responses from the two sources (expressed as percentages) [22]. Likewise, the calculation of Pneg was done in direct correspondence to the foregoing approach [22]. Third, McNemar’s test including Bonferroni adjustment was used to assess if the disagreement between the two sources of data was statistically significant [8]. A subgroup analysis was conducted to investigate whether the agreement improved when considering only individuals requiring medication for their respective conditions (data available for cardiovascular, pulmonary, and digestive tract symptoms). Furthermore, logistic regression modelling was performed to investigate if the concordance between the LSQ answers and medical survey answers was associated with specific patient or disease characteristics. The model included sex (male vs. female), age at treatment start (< 40 years vs. ≥ 40), clinical stage (stage I + II vs. III + IV), and educational level (no university degree vs. university degree). An assumption of independence among the predicting variables was made. Finally, reported late effects in the medical survey were compared in terms of LSQ status.

## Results

**Fig. 1:**
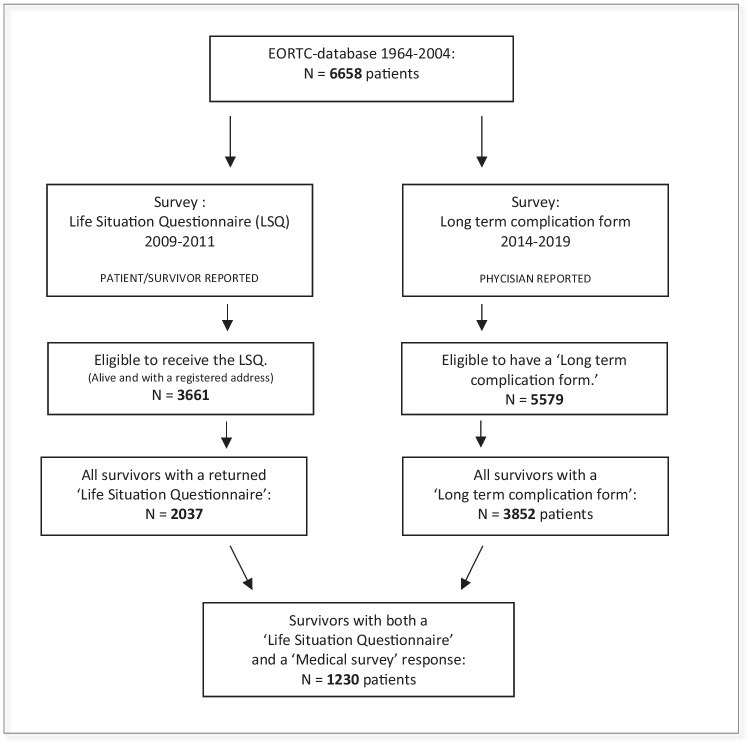
Patient flowchart

Baseline patient characteristics are shown in the supplement (supplement S2). Among the 6658 HL patients in the EORTC H1-H9 cohort, 1230 long-term HL survivors had both an LSQ and a medical survey response available and were included in this analysis (Fig. 1). The study population included both Dutch and French survivors. Of those, 49% were males and the median age at treatment start was 30 years (range 10–69 years). The median ages at completion of the LSQ and the medical survey were 47 years (range 24–84) and 53 years (range 29–91), respectively. Most survivors (86.9%) had been treated for stage I/II disease, and the majority (66.7%) had received treatment regimens involving both radiotherapy and anthracyclines (according to the H1–H9 trial designs). Almost half of the survivors (48.8%) were treated between 1994 and 2004, and the median follow-up time was 14.3 years (range 5.6–44.6 years).

**Fig. 2:**
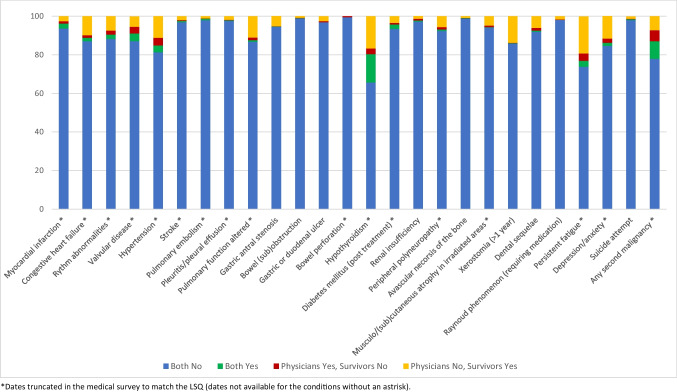
Concordance and discordance between survivors and physicians in percent (%)

Figure 2 illustrates the concordance/discordance of responses from survivors and physicians. When examining the level of agreement (based on the values of the Kappa statistic), the concordance varied across different categories (Table 1). The agreement ranged from no agreement to moderate agreement, with half of the conditions falling within the “fair” and “moderate” agreement categories. The highest kappa values were found for myocardial infarction (kappa = 0.55, 95% CI 0.43–0.66) and pulmonary embolism (kappa = 0.55, 95% CI 0.35–0.75), whereas no concordance was found for gastric or duodenal ulcers (kappa =  − 0.01, 95% CI − 0.02–[− 0.00]) and Raynaud’s phenomenon (kappa =  − 0.003, 95% CI − 0.00–[− 0.00]). The overall percentage agreement varied from 77.0% for persistent fatigue to 99.5% for bowel perforation. As the prevalence of specific late effects was rather low (less than 10% for most conditions), the overall percentage agreement was mainly driven by the agreement on the absence of symptoms. The highest Ppos was found for hypothyroidism (60.0%), and the highest Pneg for bowel perforation (99.8%).

**Table 1:** Reported late effects in the LSQ and the medical survey including summary and test statistics for concordance

	Reported in the LSQ %	Reported in the medical survey %		Kappa coefficient (95% CI)	Agreement %	Positive agreement %	Negative agreement %	McNemar’s test *P*-value	Adjusted *P*-value^
Cardiovascular									
Myocardial infarction	5.21	3.98	*	0.55 (0.43–0.66)	96.2	56.6	98.0	0.027	0.675
Congestive heart failure	12.10	2.76	*	0.22 (0.13–0.30)	89.1	25.1	94.1	< 0.001	< 0.001
Rhythm abnormalities	9.76	3.80	*	0.31 (0.21–0.40)	91.1	34.6	95.2	< 0.001	< 0.001
Valvular disease	9.34	7.32	*	0.25 (0.16–0.34)	91.2	47.8	95.2	0.051	1.00
Hypertension	14.80	7.56	*	0.25 (0.17–0.32)	84.9	32.2	91.5	< 0.001	< 0.001
Stroke	2.64	1.06	*	0.35 (0.17–0.53)	97.6	35.6	98.8	0.001	0.021
Pulmonary									
Pulmonary embolism	1.95	0.97	*	0.55 (0.35–0.75)	98.7	55.6	99.3	0.005	0.148
Pleuritis/pleural effusion	2.11	0.65	*	0.23 (0.04–0.42)	98.9	23.5	98.9	0.001	0.021
Pulmonary function altered	11.95	3.01	*	0.08 (0.02–0.15)	87.6	11.7	93.3	< 0.001	< 0.001
Digestive tract									
Gastric antral stenosis	5.37	0.16		0.03 (− 0.03–0.08)	94.6	2.9	97.2	< 0.001	< 0.001
Bowel (sub)obstruction	0.98	0.24		0.13 (− 0.10–0.36)	98.9	13.3	99.5	0.03	0.663
Bastric or duodenal ulcer	2.52	0.57		− 0.01 (− 0.02–[− 0.00])	96.9	0.0	98.4	< 0.001	0.005
Bowel perforation	0.49	0.0	*	0.0 (− 1.00–1.00)	99.5	0.0	99.8	< 0.001	0.005
Endocrine									
Hypothyroidism	31.31	17.15	*	0.48 (0.43–0.54)	80.4	60.0	87.0	< 0.001	< 0.001
Diabetes mellitus (post treatment)	5.90	3.01	*	0.51 (0.40–0.63)	95.8	53.2	97.8	< 0.001	< 0.001
Urologic									
Renal insufficiency (> doubling of s-creatinine)	1.95	1.46		0.32 (0.14–0.51)	97.7	33.3	98.8	0.35	1.00
Neurologic									
Peripheral polyneuropathy	6.34	2.03	*	0.15 (0.05–0.25)	93.1	17.6	96.4	< 0.001	< 0.001
Musculoskeletal									
Avascular necrosis of bone	1.14	0.33		0.33 (0.05–0.61)	99.0	33.3	99.5	0.01	0.23
Musculo/(sub)cutaneous atrophy in irradiated areas	5.04	1.14	*	0.09 (− 0.01–0.18)	94.4	10.5	97.1	< 0.001	< 0.001
Oral									
Xerostomia (> 1 year)	14.07	0.33		0.03 (− 0.01–0.06)	86.1	3.4	92.5	< 0.001	< 0.001
Dental sequelae	6.67	1.87		0.11 (0.019–0.20)	92.6	13.3	96.1	< 0.001	< 0.001
Miscellaneous									
Raynaud phenomenon (req. medication)	1.71	0.16		− 0.00 (− 0.00–[− 0.00])	98.1	0.0	99.1	< 0.001	0.004
Persistent fatigue	22.52	6.83	*	0.12 (0.07–0.18)	77.0	21.4	86.5	< 0.001	< 0.001
Depression/anxiety	13.41	3.74	*	0.13 (0.06–0.20)	86.1	18.4	92.4	< 0.001	< 0.001
Suicide attempt	1.63	0.73		-	-	-	-	-	-
Second malignancy									
Second malignancy	16.0	14.6	*	0.50 (0.43–0.57)	87.0	58.0	97.3	0.140	1.00

^Bonferroni-adjusted *P*-values to determine if the disagreement is statistically significant after correcting for multiple testing

*Dates in the medical survey truncated to match the LSQ (dates not available for the conditions without an asterisk)

In general, a higher prevalence of late effects was reported by the survivors. Notably, the prevalence of persistent fatigue and depression/anxiety was more than three times as high in the LSQ compared to the medical survey. In fact, the prevalence of late effects reported by physicians was consistently lower than the prevalence reported by HL survivors. Especially, subjective symptoms that cannot be directly observed or measured (e.g., xerostomia) were subjected to more extensive underreporting by physicians. The positive agreement did not significantly improve when the analyses were restricted to the subgroup of survivors requiring medication for their conditions (see supplement S3–S4).

The probability of concordance was found to be influenced by patient and disease characteristics, as revealed by logistic regression analysis (Table 2). Notably, a mixture of both positive and negative associations was observed among all predicting variables. However, there was an overall trend towards a higher concordance for survivors with higher clinical disease stage and higher educational level and those who initiated HL treatment at a younger age.

**Table 2:** Logistic regression model estimates^ (outcome = concordance between medical survey and LSQ answers)

	Sex (Ref: male)			Age at treatment start (Ref: 40 years and above)			Clinical stage (Ref: early stage)			Educational level (Ref: university degree)		
	OR	95% CI	*P*-value	OR	95% CI	*P*-value	OR	95% CI	*P*-value	OR	95% CI	*P*-value
Cardiovascular												
Myocardial infarction*	1.60	0.95–2.70	0.079	2.89	1.72–4.84	< 0.001	0.99	0.47–2.06	0.970	0.35	0.17–0.69	0.002
Congestive heart failure*	0.94	0.66–1.35	0.745	2.28	1.58–3.30	< 0.001	1.76	0.94–3.28	0.758	0.57	0.38–0.86	0.006
Rhythm abnormalities*	1.14	0.77–1.68	0.513	1.52	1.00–2.31	0.049	0.74	0.44–1.26	0.266	0.75	0.49–1.15	0.192
Valvular disease*	0.72	0.48–1.09	0.121	1.03	0.64–1.63	0.914	4.05	1.47–11.20	0.006	0.56	0.35–0.89	0.014
Hypertension*	1.14	0.83–1.56	0.418	1.91	1.37–2.67	< 0.001	1.20	0.74–1.95	0.465	0.67	0.47–0.94	0.022
Pulmonary												
Pulmonary function altered*	0.61	0.43–0.87	0.006	1.16	0.78–1.71	0.471	1.47	0.82–2.62	0.196	0.80	0.55–1.15	0.229
Digestive tract												
Gastric antral stenosis	1.28	0.77–2.13	0.342	1.36	0.78–2.37	0.277	2.36	0.84–6.60	0.102	1.06	0.62–1.80	0.832
Endocrine												
Hypothyroidism*	0.66	0.49–0.87	0.003	0.94	0.67–1.31	0.716	2.69	1.55–4.69	< 0.001	1.07	0.79–1.43	0.671
Diabetes mellitus (post treatment)*	2.72	1.50–4.93	< 0.001	4.13	2.39–7.14	< 0.001	1.22	0.53–2.78	0.642	0.63	0.33–1.17	0.144
Neurologic												
Peripheral polyneuropathy*	0.77	0.50–1.20	0.244	2.37	1.51–3.74	< 0.001	0.77	0.42–1.40	0.391	1.17	0.74–1.85	0.496
Musculoskeletal												
Musculo/(sub)cutaneous atrophy in irradiated areas*	0.51	0.30–0.87	0.013	0.57	0.28–1.14	0.109	1.40	0.59–3.32	0.440	1.39	0.84–2.31	0.195
Oral												
Xerostomia (> 1 year)	0.60	0.43–0.84	0.003	1.68	0.43–0.84	0.003	1.68	1.17–2.41	0.005	0.86	0.60–1.22	0.391
Dental sequelae	0.91	0.59–1.41	0.681	0.98	0.59–1.63	0.681	0.72	0.40–1.28	0.261	1.26	0.81–1.96	0.299
Miscellaneous												
Persistent fatigue*	0.66	0.50–0.87	0.003	1.01	0.74–1.38	0.955	0.85	0.57–1.26	0.420	0.94	0.70–1.25	0.658
Depression/anxiety*	0.74	0.53–1.03	0.074	0.80	0.53–1.19	0.272	1.31	0.77–2.24	0.316	1.14	0.81–1.60	0.454
Second malignancy												
Any second malignancy*	0.97	0.68–1.38	0.869	1.02	0.68–1.53	0.930	1.12	0.65–1.93	0.678	0.83	0.57–1.21	0.327

*OR* odds ratio

*CI* confidence interval

^Only estimates for conditions with a prevalence of more than 5% in the LSQ are shown (for all numbers, see supplement S5)

*Dates truncated in the medical survey to match the LSQ (dates not available for the conditions without an asterisk)

In a comparison of LSQ-responders versus non-responders, there was a higher percentage of long-term late effects reported by physicians in the LSQ-responder group, with significant differences for approximately half of the conditions (Table 3). Additionally, a higher proportion (59%) of LSQ non-responders were males. Also, a greater proportion of non-responders (including those who had died) had been treated in the earliest trials and had a history of relapse. No other significant differences were observed between the two groups (see supplement S2).

**Table 3:** Reported late effects in the medical survey stratified by LSQ-status

	Medical survey + LSQ *N* = 1230			Medical survey − LSQ *N* = 2275			*P*-value^
	*N* (no)	*N* (yes)	% (yes)	*N* (no)	*N* (yes)	% (yes)	
Cardiovascular							
Myocardial infarction	1169	61	5.0	2190	85	3.7	0.101
Congestive heart failure	1176	54	4.4	2214	61	2.7	**0.007**
Rhythm/conduction disturbances	1158	72	5.9	2215	60	2.6	** < 0.001**
Valvular disease	1100	130	10.6	2164	111	4.9	** < 0.001**
Stroke	1210	20	1.6	2255	20	0.9	0.065
Hypertension	1107	123	10.0	2167	108	4.7	** < 0.001**
Pulmonary							
Pulmonary embolism	1214	16	1.3	2252	23	1.0	0.544
Pleuritis/pleural effusion	1218	12	1.0	2249	26	1.1	0.772
Pulmonary function altered (NOS)	1213	17	1.4	2251	24	1.1	0.480
Functional test altered (NOS)	1210	20	1.6	2262	13	0.6	**0.003**
- Restrictive	1213	17	1.4	2259	16	0.7	0.071
- Obstructive	1216	14	1.1	2266	9	0.4	**0.016**
Digestive tract							
Bowel (sub)obstruction	1227	3	0.2	2270	5	0.2	1.000
Bowel perforation	1230	0	0.0	2273	2	0.1	0.544
Gastric antral stenosis	1228	2	0.2	2275	0	0.0	0.123
Peptic ulcer (NOS)	1228	2	0.2	2273	2	0.1	0.616
Gastric ulcer	1227	3	0.2	2270	5	0.2	1.00
Duodenal ulcer	1226	4	0.3	2275	0	0.0	**0.015**
Endocrine							
Hypothyroidism	994	236	19.2	2073	202	8.9	** < 0.001**
Diabetes mellitus (post treatment)	1187	43	3.5	2222	53	2.3	**0.049**
Urologic							
Renal insufficiency (> doubling of s-creatinine)	1212	18	1.5	2254	21	0.9	0.192
Neurologic							
Peripheral polyneuropathy	1198	32	2.6	2231	44	1.9	0.229
Musculoskeletal							
Avascular necrosis of bone	1226	4	0.3	2265	10	0.4	0.782
Musculo/(sub)cutaneous atrophy in irradiated areas	1207	23	1.9	2263	12	0.5	** < 0.001**
Oral							
Dental prosthesis	1221	9	0.7	2265	10	0.4	0.374
Dental sequelae	1222	8	0.7	2263	13	0.6	0.950
Xerostomia (> 1 year)	1226	4	0.3	2266	9	0.4	1.00
Miscellaneous							
Raynaud phenomenon	1228	2	0.2	2268	7	0.3	0.508
Depression/anxiety	1172	58	4.7	2218	57	2.5	** < 0.001**
Persistent fatigue	1128	102	8.3	2159	116	5.1	** < 0.001**
- Female	560	67	10.7	886	46	4.9	** < 0.001**
- Male	568	35	5.8	1273	70	5.2	0.647
Suicide attempt	1221	9	0.7	2267	8	0.4	0.132
Second malignancy							
Any second malignancy	978	252	20.5	1936	339	14.9	** < 0.001**

The two-proportion *z*-test is used to compare the two observed proportions (= 2-sample test for equality of proportions with continuity correction). Fisher’s exact test is used when expected frequencies are below 5

## Discussion

To the best of our knowledge, there are no other studies comparing the reporting of late effects by HL survivors to that by physicians. Hence, this study offers a novel and unprecedented understanding of survivorship within this population. We found that the prevalence of patient-reported late effects repeatedly exceeded that reported by physicians and, at best, the level of agreement was found to be moderate. Also, a higher percentage of reported late effects was observed among those who responded to the LSQ compared to the non-responder group.

The observed differences in reporting between survivors and physicians, as well as those between LSQ responders and non-responders, could hold significant implications for survivorship care and the development of survivorship guidelines. Consequently, the presented data calls for a critical reflection on the potential for an erroneous estimation of the true prevalence of long-term consequences of HL treatment. An overestimation could result in unnecessary interventions or heightened concerns for patients, potentially impacting their quality of life. Conversely, an underestimation could result in inadequate monitoring or support for individuals dealing with adverse effects of HL treatment.

Several factors may contribute to the observed differences between survivors and physicians. In this cohort, no structured follow-up for evaluation of long-term toxicity existed. Unstructured clinical visits may not have captured all symptoms, especially those that survivors do not explicitly mention during their appointments [8]. Supporting this, Homsi et al. showed in a palliative setting that the median number of symptoms identified using systematic assessment was tenfold higher than that volunteered by patients without prompting [25]. Also, because of limited time during patient visits, less attention may be paid to subjective symptoms, especially those with no treatment options or those mild in severity [2]. Likewise, patients may be unaware that treatment options of certain symptoms (e.g., dry mouth) exist and therefore do not mention them [25], or they refrain from reiterating their concerns if they have been addressed during earlier visits. Conversely, physicians could decide not to report a symptom if they judged it unrelated to treatment [2]. Thus, unstructured documentation of late effects can lead to incomplete medical records.

In line with other findings, conditions related to emotional well-being (e.g., depression and anxiety) and more diffuse symptoms (e.g., persistent fatigue) were reported far more often by the survivors than by the physicians [7, 8, 26, 27]. A possible explanation could be that the predominant focus of oncology/hematology visits tends to be on biological treatment and symptom management [28]. Physicians may prioritize addressing physical symptoms directly related to the disease, potentially overlooking psychological and less tangible conditions. Consequently, these symptoms may not receive adequate attention during clinical evaluations, contributing to the underreporting observed by physicians. Moreover, it has been shown that less than one in four patients with psychological symptoms spontaneously disclose them during their medical appointment due to concerns about burdening the health care professionals or the fear of being stigmatized [28, 29], further contributing to the discrepancy in reporting.

Considering the above, the observations and interpretations of late effects made by physicians may be influenced by many factors. However, when shifting the focus to the reliance on self-reported outcomes, another significant concern arises—the potential risk for misclassification of symptoms [30]. An illustrative example of discrepancies in survivor reporting in this study is LSQ responders who responded negatively to the presence of hypertension, while simultaneously confirming the use of antihypertensive medication. It could therefore be questioned whether the questionnaire was formulated well enough to adequately capture the necessary information. In all circumstances, this incongruity raises questions about the accuracy or reliability of the self-reported information as data has not been externally validated.

Previous concordance research exploring other areas of physician vs. patient-reports has reported mixed results across a range of disciplines [4, 9, 31–35]. In a systematic review by Atkinson et al., most studies proved poor to moderate agreement in symptom reporting [36] which aligns with our findings where the kappa ranged from < 0 to 0.52. The Childhood Cancer Survivorship Study (CCSS) provides a possible explanation for the observed discrepancy between self-reported late effects and medical records, highlighting the influence of diagnostic criteria on concordance. Specifically, it reveals higher agreement for conditions with clear diagnostic criteria and lower agreement for those with less established criteria [37]. Similar observations were made in a study by Louie et al. who validated self-reported complications by bone marrow transplantation survivors [38]. Hence, an unintentional tendency towards overreporting by survivors for conditions with less established diagnostic criteria may be suspected. These observations are in line with the findings of our study, where conditions of a more critical nature that typically require hospital treatment (such as heart failure and myocardial infarction) are presumed to be accurately documented in medical records. Nevertheless, these same conditions were reported more frequently by the HL survivors.

The CCSS emphasizes the intricate interplay between diagnostic criteria and concordance and sheds light on one of the challenges in assessing and documenting late effects in survivorship research. Additionally, socio-demographic and disease-related factors may play a significant role [9, 39]. For instance, the trend towards a higher concordance among survivors with more advanced disease stages at time of diagnosis could indicate that exposure to more aggressive treatment regimens potentially results in more pronounced and noticeable late effects and, therefore, better concordance. Another plausible explanation could be that physicians anticipate a higher occurrence of late effects in patients with advanced disease and, thus, are more attuned to recognize and discuss them. Our study also identified a trend towards a lower concordance among survivors with lower educational level and higher age at treatment start which raises some interesting questions. Are survivors with a higher educational level better at expressing and articulating their symptoms (leading to a more accurate representation of their late effects) or do they find it more difficult to accept “the price to pay”? And do younger patients have different expectations or priorities compared to older patients? Although not statistically significant and primarily exploratory in nature, these results might reflect varying perceptions of late effects, differences in communication style, and patient empowerment—factors worth noticing.

The utilization of patient-reported outcomes in medical research and healthcare interventions is valuable, yet it comes with inherent limitations that must be considered. One significant concern revolves around the potential influence of non-response bias. In this study, a higher percentage of non-responders (including those who had died) was treated in the earliest EORTC HL trials and therefore received more radiotherapy as single treatment modality. Likewise, there was a higher percentage of the non-responders who experienced a relapse and, consequently, were expected to suffer more late effects. However, this potential bias does not pose a significant concern other than our estimates being a bit conservative. Conversely, a higher percentage of reported late effects was observed in the LSQ-responders’ group, which could diminish the representativeness of the study cohort. This observation is particularly noteworthy as it unveils a previously unexplored phenomenon: individuals experiencing fewer or less significant late effects may be less inclined to participate in questionnaire-based surveys, as evidenced by the higher reported percentage of late effects among LSQ responders. This disparity underscores the significant influence of assessment methods on identifying and documenting late effects, which holds paramount importance, especially in studies like the CCSS that heavily rely on self-reported data. Also, similar to other studies [40], we found a disproportionate representation of females among the LSQ responders. This introduces another layer of complexity, as differences in health-seeking behavior or communication styles between the sexes may influence the reported outcomes [41]. Furthermore, the two surveys were not developed to make a direct comparison and varying terminology between the LSQ, and the medical survey exists. Consequently, survivors and physicians might interpret the questions differently, leading to discrepancies in their responses. Moreover, the survivor-reported symptoms collected through the LSQ could be affected by recall bias.

Relying solely on either physician- or survivor-reported outcomes may not provide a comprehensive understanding of the complex and multifaceted nature of long-term late effects in this group. Despite non-responders to the LSQ having fewer late effects registered in the medical survey, the observed discrepancies still indicate that symptoms are being overlooked. Therefore, perspectives from both survivors and physicians should be considered. Leveraging data from both sources offers not only a more nuanced picture of the survivors’ health but also contributes to a more personalized survivorship care planning. However, researchers and healthcare professionals must approach patient-reported outcome data with caution, acknowledging and addressing the inherent limitations to ensure the robustness and applicability of the findings. Nonetheless, the incorporation of survivor-reported outcomes into the design of prospective HL trials should be considered as an obligatory part in advancing knowledge in the field of HL survivorship care.

## Conclusion

In this study, substantial underreporting of late effects by physicians was observed, especially in the context of subjective conditions which are not easily quantified. However, the interpretation of these findings warrants consideration of potential biases stemming from differential participation, with those experiencing more (severe) late effects being more likely to respond to surveys. While this may mitigate some of the observed discrepancies, our data highlight a group of survivors whose needs may be overlooked. Integrating perspectives from both survivors and physicians is therefore essential to enhance our understanding of late effects and improve the quality of care for long-term HL survivors.

## Supplementary Information

Below is the link to the electronic supplementary material.

Supplementary file1 (DOCX 129 KB)

## Data Availability

Data shall be shared according to the EORTC data release policy (https://www.eortc.org/data-sharing/).
