# Supplementary material for: Concordance between late effects reported by physicians and patients in a cohort of long-term Hodgkin lymphoma survivors: an analysis of data from nine consecutive EORTC-LYSA trials
Source: J Cancer Surviv. 2024 Oct 18;20(3):883–94. doi: 10.1007/s11764-024-01694-0 (PMC13144188; doi:10.1007/s11764-024-01694-0)
Supplement: Supplementary file 1 — Supplementary file1 (DOCX 129 KB) [file 11764_2024_1694_MOESM1_ESM.docx]

**Supplementary**

- Supplement S1. EORTC MEDICAL UPDATE overview of data collection and content.
- Supplement S2: Characteristics of the study population with treatment information available (stratified).
- Supplement S3: Subgroup analyses (restricted to those who requires medication).
- Supplement S4: Subgroup analyses (dates truncated + medication only).
- Supplement S5. Logistic regression model estimates (outcome = concordance between medical survey and LSQ answers).
- Supplement S6: Analyses of agreement (2x2 table for each of the included conditions).
- Supplement S7: Analyses of agreement stratified by sex (2x2 table for each of the included conditions).
- Supplement S8: Cohens Kappa coefficients according to (sub)group.

**Corresponding author**: [sidsel.jacobsen.juul@regionh.dk](mailto:sidsel.jacobsen.juul@regionh.dk)

**S1. EORTC MEDICAL UPDATE** overview of data collection and content

A part of the EORTC survivorship strategy encompasses a comprehensive initiative aimed at gathering data related to late medical sequelae after treatment for Hodgkin lymphoma.

Data originates from the EORTC Lymphoma Group, a subgroup within the EORTC focusing on Hodgkin lymphoma (HL). The group has a history of conducting clinical trials dating back to 1964. Since 1993, these trials have been carried out in collaboration with the Lymphoma Study Association (LYSA), former known as Groupe d’Étude des Lymphomes de l’adulte (GELA).

The first nine trials (H1-H9) were conducted over a span of four decades (1964-2004) and a total of 6658 patients from 13 European countries (BE ⋅ CH ⋅ DE ⋅ ES ⋅ FR ⋅ GB ⋅ HR ⋅ IT ⋅ NL ⋅ PL ⋅ PT ⋅ SI ⋅ SK) were randomized.

Data collection for this part of the survivorship strategy (medical update) took place through the administration of a survey named “EORTC-GELA survey of patients enrolled in phase III trials on Hodgkin’s lymphoma”.

The EORTC-GELA survey was designed for physicians to report on late medical toxicities following HL treatment and was distributed to all H1-H9 participating centres where a principal investigator was available. Centres in the Netherlands, France, Croatia, Slovak Republic, and Poland participated.

The survey forms were sent out prefilled with data based on information already available in existing files. The medical professionals (or data managers) responsible for filling in the survey then had the option to review and make corrections to any event that they believed was recorded incorrectly.

The survey was completed between 2014-2019 and consisted of the following components:

1. Identification Form
2. Progression/relapse Form
3. Long term complication Form
4. Second malignancy Form
5. Last news Form (survival status)

Once completed, the survey forms were sent to the Lymphoma Group data manager at the EORTC headquarters in Brussel.

The overall response rate from the participating centres was 69%.

**S2**: Characteristics of the EORTC H1-H9 study cohort with treatment information available (stratified by survey status)

|  |  | |  | |  | |  | |  | |  | |  | |  | |
| --- | --- | --- | --- | --- | --- | --- | --- | --- | --- | --- | --- | --- | --- | --- | --- | --- |
|  |  | | **EORTC H1 – H9 cohort**  (total) | | **EORTC H1 – H9 cohort**  w/ Medical survey | | **EORTC H1 – H9 cohort**  w/o Medical survey | | **EORTC H1 – H9 cohort**  w/ LSQ (Alive and response) | | **EORTC H1 – H9 cohort**  w/o LSQ (Dead or no response) | | **Medical survey**  **w/ LSQ** (Response in both) | | **Medical survey**  **w/o LSQ** | |
|  |  | |  |  |  | |  | |  | |  | |  | |  | |
|  |  | | N | (%) | N | (%) | N | (%) | N | (%) | N | (%) | N | (%) | N | (%) |
|  |  | |  |  |  | |  | |  | |  | |  | |  | |
|  |  | |  |  |  | |  | |  | |  | |  | |  | |
| **Total no. of patients** | | | 6039 | 100 | 3505 | 100 | 2534 | 100 | 1919 | 100 | 4120 | 100 | 1230 | 100 | 2275 | 100 |
| **Sex** | | |  |  |  |  |  |  |  |  |  |  |  |  |  |  |
|  | Male | | 3274 | 54.2 | 1946 | 55.5 | 1328 | 52.4 | 938 | 48.9 | 2336 | 56.7 | 603 | 49.0 | 1343 | 59.0 |
|  | Female | | 2765 | 45.8 | 1559 | 44.5 | 1206 | 47.6 | 981 | 51.1 | 1784 | 43.3 | 627 | 51.0 | 932 | 41.0 |
| **Country** | | |  |  |  |  |  |  |  |  |  |  |  |  |  |  |
|  | The Netherlands | | 2209 | 36.6 | 2062 | 58.8 | 147 | 5.8 | 895 | 46.6 | 1314 | 31.9 | 841 | 68.4 | 1221 | 53.7 |
|  | France | | 2682 | 44.4 | 1425 | 40.7 | 1257 | 49.6 | 722 | 37.6 | 1960 | 47.6 | 388 | 31.5 | 1037 | 45.6 |
|  | Belgium | | 630 | 10.4 | 0 | 0.0 | 630 | 24.9 | 150 | 7.8 | 480 | 11.7 | 0 | 0.0 | 0 | 0.0 |
|  | Croatia | | 16 | 0.3 | 15 | 0.4 | 1 | 0.03 | 0 | 0.0 | 16 | 0.4 | 0 | 0.0 | 15 | 0.7 |
|  | Germany | | 23 | 0.4 | 0 | 0.0 | 23 | 0.9 | 0 | 0.0 | 23 | 0.6 | 0 | 0.0 | 0 | 0.0 |
|  | Italy | | 218 | 3.6 | 0 | 0.0 | 218 | 8.6 | 90 | 4.7 | 128 | 3.1 | 0 | 0.0 | 0 | 0.0 |
|  | Poland | | 65 | 1.1 | 1 | 0.02 | 64 | 2.5 | 0 | 0.0 | 65 | 1.6 | 0 | 0.0 | 1 | 0.04 |
|  | Portugal | | 38 | 0.6 | 0 | 0.0 | 38 | 1.5 | 0 | 0.0 | 38 | 0.9 | 0 | 0.0 | 0 | 0.0 |
|  | Slovak Republic | | 50 | 0.8 | 2 | 0.1 | 48 | 1.9 | 29 | 1.5 | 21 | 0.5 | 1 | 0.1 | 1 | 0.04 |
|  | Slovenia | | 76 | 1.3 | 0 | 0.0 | 76 | 3.0 | 27 | 1.4 | 49 | 1.2 | 0 | 0.0 | 0 | 0.0 |
|  | Spain | | 8 | 0.1 | 0 | 0.0 | 8 | 0.3 | 0 | 0.0 | 8 | 0.2 | 0 | 0.0 | 0 | 0.0 |
|  | Switzerland | | 13 | 0.2 | 0 | 0.0 | 13 | 0.5 | 6 | 0.3 | 7 | 0.2 | 0 | 0.0 | 0 | 0.0 |
|  | United Kingdom | | 10 | 0.2 | 0 | 0.0 | 10 | 0.4 | 0 | 0.0 | 10 | 0.2 | 0 | 0.0 | 0 | 0.0 |
|  | Missing | | 1 | 0.02 | 0 | 0.0 | 1 | 0.03 | 0 | 0.0 | 1 | 0.02 | 0 | 0.0 | 0 | 0.0 |
| **Age at treatment start** | | |  |  |  |  |  |  |  |  |  | |  |  |  |  |
|  | Years | | Median: 30.0  IQR: 23.0 - 40.0  Range: 5.0 - 73.0 | | Median: 30.0  IQR: 23.0 - 40.0  Range: 5.0 - 73.0 | | Median: 30.0  IQR: 23.0 - 41.0  Range: 7.0 - 70.0 | | Median: 29.0  IQR: 23.0 - 39.0  Range: 10.0 - 69.0 | | Median: 30.0  IQR: 23.0 - 41.0  Range: 5.0-73.0 | | Median: 30.0  IQR: 22.0 - 40.0  Range: 10.0 - 69.0 | | Median: 30.0  IQR: 23.0 - 41.0  Range: 5.0 - 73.0 | |
|  |  | |  |  |  |  |  |  |  |  |  |  |  |  |  | |
|  | < 20 years | | 719 | 11.9 | 438 | 12.5 | 281 | 11.1 | 235 | 12.2 | 484 | 11.7 | 155 | 12.6 | 283 | 12.4 |
|  | [20–30] years | | 2388 | 39.5 | 1380 | 39.4 | 1008 | 39.8 | 797 | 41.5 | 1591 | 38.6 | 488 | 39.7 | 892 | 39.2 |
|  | (30–40] years | | 1427 | 23.6 | 821 | 23.4 | 606 | 23.9 | 468 | 24.4 | 659 | 16.0 | 302 | 24.6 | 519 | 22.8 |
|  | (40–50] years | | 827 | 13.7 | 475 | 13.6 | 352 | 13.9 | 276 | 14.4 | 551 | 13.4 | 189 | 15.4 | 286 | 12.6 |
|  | (50–60] years | | 438 | 7.3 | 247 | 7.0 | 191 | 7.5 | 107 | 5.6 | 331 | 8.0 | 75 | 6.1 | 172 | 7.6 |
|  | (60–70] years | | 235 | 3.9 | 140 | 3.9 | 95 | 3.7 | 36 | 1.9 | 199 | 4.8 | 21 | 1.7 | 119 | 5.2 |
|  | >70 years | | 3 | 0.04 | 3 | 0.1 | 0 | 0.0 | 0 | 0.0 | 3 | 0.1 | 0 | 0.0 | 3 | 0.1 |
|  | Missing | | 2 | 0.03 | 1 | 0.02 | 1 | 0.03 | 0 | 0.0 | 2 | 0.04 | 0 | 0.0 | 1 | 0.04 |
| **Clinical stage** | | |  |  |  |  |  |  |  |  |  |  |  |  |  |  |
|  | I | | 1561 | 25.8 | 980 | 28.0 | 581 | 22.9 | 467 | 24.3 | 1094 | 26.6 | 317 | 25.8 | 663 | 29.1 |
|  | II | | 3570 | 59.1 | 2012 | 57.4 | 1558 | 61.5 | 1183 | 61.6 | 2387 | 57.9 | 752 | 61.1 | 1260 | 55.4 |
|  | III-IV | | 906 | 15.0 | 511 | 14.6 | 395 | 15.6 | 268 | 14.0 | 638 | 15.5 | 160 | 13.0 | 351 | 15.4 |
|  | Missing | | 0 | 0.0 | 2 | 0.1 | 0 | 0.0 | 1 | 0.1 | 1 | 0.1 | 1 | 0.1 | 1 | 0.04 |
| **Risk categorisation** | | |  |  |  |  |  |  |  |  |  |  |  |  |  |  |
|  | Early stage (stage I and II) | |  |  |  |  |  |  |  |  |  |  |  |  |  |  |
|  | - Favourable | | 1886 | 36.8 | 1086 | 36.3 | 800 | 37.4 | 678 | 41.1 | 1208 | 34.7 | 443 | 41.4 | 643 | 33.4 |
|  | - Unfavourable | | 2625 | 51.2 | 1484 | 49.6 | 1141 | 53.3 | 842 | 51.0 | 1783 | 51.2 | 541 | 50.6 | 943 | 49.0 |
|  | - Missing | | 620 | 12.1 | 422 | 14.1 | 198 | 9.3 | 130 | 7.9 | 490 | 14.1 | 85 | 8.0 | 337 | 17.5 |
|  | Advanced stage  (stage III and IV) | |  |  |  |  |  |  |  |  |  |  |  |  |  |  |
|  | - IPS 0-2 | | 45 | 5.0 | 20 | 3.9 | 25 | 6.3 | 18 | 6.7 | 27 | 4.2 | 7 | 4.4 | 13 | 3.7 |
|  | - IPS 3-7 | | 312 | 34.4 | 198 | 38.7 | 114 | 28.9 | 68 | 25.4 | 244 | 38.3 | 54 | 33.7 | 144 | 41.0 |
|  | - Missing | | 549 | 60.6 | 293 | 57.3 | 256 | 64.8 | 182 | 67.9 | 367 | 57.5 | 99 | 61.9 | 194 | 55.3 |
| **Histology** | | |  |  |  |  |  |  |  |  |  |  |  |  |  |  |
|  | Nodular sclerosis | | 4424 | 73.3 | 2601 | 74.2 | 1823 | 71.9 | 1488 | 77.5 | 2936 | 71.3 | 966 | 78.5 | 1635 | 71.9 |
|  | Mixed cellularity | | 1018 | 16.9 | 598 | 17.1 | 420 | 16.6 | 258 | 13.4 | 760 | 18.4 | 158 | 12.8 | 440 | 19.3 |
|  | Lymphocyte depleted | | 56 | 0.9 | 31 | 0.9 | 25 | 1.0 | 14 | 0.7 | 42 | 1.0 | 8 | 0.7 | 23 | 1.0 |
|  | Lymphocyte rich | | 0 | 0.0 | 0 | 0.0 | 0 | 0.0 | 0 | 0.0 | 0 | 0.0 | 0 | 0.0 | 0 | 0.0 |
|  | Lymphocyte predominant | | 332 | 5.5 | 184 | 5.2 | 148 | 5.8 | 98 | 5.1 | 234 | 5.7 | 63 | 5.2 | 121 | 5.3 |
|  | Missing | | 209 | 3.5 | 91 | 2.6 | 118 | 4.7 | 61 | 3.2 | 148 | 3.6 | 35 | 2.8 | 56 | 2.5 |
| **Period of treatment start** | | |  |  |  |  |  |  |  |  |  |  |  |  |  |  |
|  | 1964–1973 | | 294 | 4.9 | 201 | 5.7 | 93 | 3.7 | 44 | 2.3 | 250 | 6.1 | 22 | 1.8 | 179 | 7.9 |
|  | 1974–1982 | | 702 | 11.6 | 514 | 14.7 | 188 | 7.4 | 127 | 6.6 | 575 | 14.0 | 100 | 8.1 | 414 | 18.2 |
|  | 1983–1995 | | 2546 | 42.2 | 1506 | 43.0 | 1040 | 41.0 | 773 | 40.3 | 1773 | 43.0 | 513 | 41.7 | 993 | 43.6 |
|  | 1996–2004 | | 2497 | 41.3 | 1284 | 36.6 | 1213 | 47.9 | 975 | 50.8 | 1522 | 36.9 | 595 | 48.4 | 689 | 30.3 |
|  | Missing | | 0 | 0.0 | 0 | 0.0 | 0 | 0.0 | 0 | 0.0 | 0 | 0.0 | 0 | 0.0 | 0 | 0.0 |
| **Primary treatment** | | |  |  |  |  |  |  |  |  |  |  |  |  |  |  |
|  | No treatment | | 90 | 1.5 | 34 | 1.0 | 56 | 2.2 | 21 | 1.1 | 69 | 1.7 | 10 | 0.8 | 24 | 1.1 |
|  | RT alone | | 1378 | 22.8 | 924 | 26.4 | 454 | 17.9 | 337 | 17.6 | 1041 | 25.3 | 234 | 19.0 | 690 | 30.3 |
|  | CT alone | | 678 | 11.2 | 347 | 9.9 | 331 | 13.1 | 168 | 8.8 | 510 | 12.4 | 95 | 7.7 | 252 | 11.1 |
|  | RT + CT | | 3893 | 64.5 | 2200 | 62.8 | 1693 | 66.8 | 1393 | 72.6 | 2500 | 60.7 | 891 | 72.4 | 1309 | 57.5 |
|  | Missing | | 0 | 0.0 | 0 | 0.0 | 0.0 | 0.0 | 0 | 0.0 | 0 | 0.0 | 0 | 0.0 | 0 | 0.0 |
| **Any relapse** | | |  |  |  |  |  |  |  |  |  |  |  |  |  |  |
|  | Yes | | 1112 | 18.4 | 735 | 21.0 | 377 | 14.9 | 190 | 9.9 | 922 | 22.4 | 127 | 10.3 | 608 | 26.7 |
|  | No | | 4927 | 81.6 | 2770 | 79.0 | 2157 | 85.1 | 1729 | 90.1 | 3198 | 77.6 | 1103 | 89.7 | 1667 | 73.3 |
|  | Missing | | 0 | 0.0 | 0 | 0.0 | 0 | 0.0 | 0 | 0.0 | 0 | 0.0 | 0 | 0.0 | 0 | 0.0 |
| **Trial** | | |  |  |  |  |  |  |  |  |  |  |  |  |  |  |
|  | H1 | | 230 | 3.8 | 150 | 4.3 | 80 | 3.2 | 35 | 1.8 | 195 | 4.7 | 15 | 1.2 | 135 | 5.9 |
|  | H2 | | 199 | 3.3 | 166 | 4.7 | 33 | 1.3 | 31 | 1.6 | 168 | 4.0 | 28 | 2.3 | 138 | 6.1 |
|  | H5 | | 477 | 7.9 | 350 | 10.0 | 127 | 5.0 | 88 | 4.6 | 389 | 9.4 | 68 | 5.5 | 282 | 12.4 |
|  | H3B4 | | 205 | 3.4 | 122 | 3.5 | 83 | 3.3 | 33 | 1.7 | 172 | 4.2 | 19 | 1.5 | 103 | 4.5 |
|  | H6 | | 532 | 8.8 | 384 | 11.0 | 148 | 5.8 | 154 | 8.0 | 378 | 9.2 | 120 | 9.8 | 264 | 11.6 |
|  | H7 | | 757 | 12.5 | 460 | 13.1 | 297 | 11.7 | 236 | 12.3 | 521 | 12.6 | 160 | 13.0 | 300 | 13.2 |
|  | H3-4 | | 708 | 11.7 | 393 | 11.2 | 315 | 12.4 | 237 | 12.4 | 471 | 11.4 | 143 | 11.6 | 250 | 10.9 |
|  | H8 | | 1439 | 23.8 | 733 | 20.9 | 706 | 27.9 | 482 | 25.1 | 957 | 23.2 | 313 | 25.4 | 420 | 18.5 |
|  | H9 | | 1492 | 24.7 | 747 | 21.3 | 745 | 29.4 | 623 | 32.5 | 869 | 21.1 | 364 | 29.6 | 383 | 16.8 |
|  |  |  |  |  |  | |  | |  | |  | |  | |  | |

**S3**. Subgroup analyses (those who requires medication only)

|  | |  |  |  |  |  |  |  |  |
| --- | --- | --- | --- | --- | --- | --- | --- | --- | --- |
|  | | **Reported in the**  **LSQ %** | **Reported in the**  **Medical survey %** | **Kappa coefficient**  **(95% CI)** | **Agreement %** | **Positive agreement %** | **Negative agreement %** | **McNemar’s**  **Test P-value** | **Adjusted**  **P-value^** |
|  | |  |  |  |  |  |  |  |  |
|  | |  |  |  |  |  |  |  |  |
|  | |  |  |  |  |  |  |  |  |
| **Cardiovascular** | |  |  |  |  |  |  |  |  |
|  | Myocardial infarction |  |  |  |  |  |  |  |  |
|  | - Medication (Yes) | NA | - | - | - | - | - | - | - |
|  | Congestive heart failure |  |  |  |  |  |  |  |  |
|  | - Medication (Yes) | 9.76 | 2.20 | 0.16 (0.08-0.24) | 90.3 | 19.1 | 94.9 | <0.001 | <0.001 |
|  | Rhythm abnormalities |  |  |  |  |  |  |  |  |
|  | - Medication (Yes) | 7.24 | 3.09 | 0.30 (0.19-0.41) | 93.1 | 33.1 | 96.4 | <0.001 | 0.001 |
|  | Valvular disease |  |  |  |  |  |  |  |  |
|  | - Medication (Yes) | 5.04 | 4.23 | 0.26 (0.15-0.38) | 93.5 | 29.8 | 96.6 | 0.314 | 1.00 |
|  | Hypertension |  |  |  |  |  |  |  |  |
|  | - Medication (Yes) | 14.88 | 6.99 | 0.17 (0.10-0.24) | 83.5 | 24.5 | 90.7 | <0.001 | <0.001 |
|  | Stroke |  |  |  |  |  |  |  |  |
|  | - Medication (Yes) | 1.63 | 0.81 | 0.12 (-0.04-0.29) | 97.9 | 13.3 | 98.9 | 0.078 | 1.00 |
| **Pulmonary** | |  |  |  |  |  |  |  |  |
|  | Pulmonary embolism |  |  |  |  |  |  |  |  |
|  | - Medication (Yes) | 1.87 | 0.89 | 0.46 (0.26-0.67) | 98.5 | 47.1 | 99.3 | 0.009 | 0.24 |
|  | Pleuritis/pleural effusion |  |  |  |  |  |  |  |  |
|  | - Medication (Yes) | 1.87 | 0.57 | 0.06 (-0.07-0.18) | 97.7 | 6.7 | 98.9 | 0.005 | 0.11 |
|  | Pulmonary function altered |  |  |  |  |  |  |  |  |
|  | - Medication (Yes) | 7.07 | 0.65 | 0.05 (-0.01-0.12) | 92.8 | 6.3 | 96.2 | <0.001 | <0.001 |
| **Digestive tract** | |  |  |  |  |  |  |  |  |
|  | Gastric antral stenosis |  |  |  |  |  |  |  |  |
|  | - Medication (Yes) | 3.33 | 0.16 | 0.04 (-0.04-0.13) | 96.7 | 4.7 | 98.3 | <0.001 | <0.001 |
|  | Bowel (sub)obstruction |  |  |  |  |  |  |  |  |
|  | - Medication (Yes) | 0.65 | 0.08 | -0.001 (-0.004-0.0011) | 99.3 | 0.0 | 99.6 | 0.046 | 1.00 |
|  | Gastric or duodenal ulcer |  |  |  |  |  |  |  |  |
|  | - Medication (Yes) | 2.76 | 0.33 | -0.006 (-0.011-[-1.00]) | 96.9 | 0.0 | 98.4 | <0.001 | <0.001 |
|  | Bowel perforation |  |  |  |  |  |  |  |  |
|  | - Medication (Yes) | 0.49 | 0.0 | 0.0 (-1.00-1.00) | 99.5 | 0.0 | 99.8 | <0.001 | <0.001 |
|  | |  |  |  |  |  |  |  |  |

^Bonferroni-adjusted significance level to determine if the disagreement is statistically significant after multiple testing.

*Data (medication yes vs. no) not available for endocrine, urologic, neurologic, musculoskeletal, oral, miscellaneous, and second malignancy.

**S4**. Subgroup analyses (those who requires medication only + dates truncated in the medical survey, so they match the LSQ)

|  | |  |  |  |  |  |  |  |  |
| --- | --- | --- | --- | --- | --- | --- | --- | --- | --- |
|  | | **Reported in the LSQ %** | **Reported in the**  **Medical survey %** | **Kappa coefficient**  **(95% CI)** | **Agreement %** | **Positive agreement %** | **Negative agreement %** | **McNemar’s**  **Test P-value** | **Adjusted**  **P-value^** |
|  | |  |  |  |  |  |  |  |  |
|  | |  |  |  |  |  |  |  |  |
|  | |  |  |  |  |  |  |  |  |
| **Cardiovascular** | |  |  |  |  |  |  |  |  |
|  | Myocardial infarction |  |  |  |  |  |  |  |  |
|  | - Medication (Yes) | NA | - | - | - | - | - | - | - |
|  | Congestive heart failure |  |  |  |  |  |  |  |  |
|  | - Medication (Yes) | 9.76 | 1.22 | 0.14 (0.06-0.22) | 91.0 | 15.5 | 95.2 | <0.001 | <0.001 |
|  | Rhythm abnormalities |  |  |  |  |  |  |  |  |
|  | - Medication (Yes) | 7.24 | 2.68 | 0.32 (0.21-0.43) | 93.4 | 34.7 | 96.6 | <0.001 | <0.001 |
|  | Valvular disease |  |  |  |  |  |  |  |  |
|  | - Medication (Yes) | 5.04 | 3.17 | 0.28 (0.16-0.40) | 94.4 | 30.9 | 97.1 | 0.028 | 0.697 |
|  | Hypertension |  |  |  |  |  |  |  |  |
|  | - Medication (Yes) | 14.88 | 5.93 | 0.18 (0.11-0.25) | 84.3 | 25.1 | 91.2 | <0.001 | <0.001 |
|  | Stroke |  |  |  |  |  |  |  |  |
|  | - Medication (Yes) | 1.63 | 0.48 | 0.15 (-0.04-0.34) | 98.2 | 15.4 | 99.1 | 0.006 | 0.139 |
| **Pulmonary** | |  |  |  |  |  |  |  |  |
|  | Pulmonary embolism |  |  |  |  |  |  |  |  |
|  | - Medication (Yes) | 1.87 | 0.65 | 0.51 (0.30-0.73) | 98.8 | 51.6 | 99.4 | <0.001 | 0.008 |
|  | Pleuritis/pleural effusion |  |  |  |  |  |  |  |  |
|  | - Medication (Yes) | 1.87 | 0.32 | 0.07 (-0.07-0.20) | 97.9 | 7.4 | 98.9 | <0.001 | 0.008 |
|  | Pulmonary function altered |  |  |  |  |  |  |  |  |
|  | - Medication (Yes) | 7.07 | 0.56 | 0.04 (-0.02-0.09) | 92.9 | 4.4 | 96.3 | <0.001 | <0.001 |
| **Digestive tract** | |  |  |  |  |  |  |  |  |
|  | Gastric antral stenosis |  |  |  |  |  |  |  |  |
|  | - Medication (Yes) | - | NA | - | - | - | - | - | - |
|  | Bowel (sub)obstruction |  |  |  |  |  |  |  |  |
|  | - Medication (Yes) | - | NA | - | - | - | - | - | - |
|  | Gastric or duodenal ulcer |  |  |  |  |  |  |  |  |
|  | - Medication (Yes) | - | NA | - | - | - | - | - | - |
|  | Bowel perforation |  |  |  |  |  |  |  |  |
|  | - Medication (Yes) | 0.49 | 0.0 | 0.0 (-1.00-1.00) | 99.5 | 0.0 | 99.8 | <0.001 | <0.001 |
|  | |  |  |  |  |  |  |  |  |

^Bonferroni-adjusted significance level to determine if the disagreement is statistically significant after multiple testing.

*Data (medication yes vs. no) not available for endocrine, urologic, neurologic, musculoskeletal, oral, miscellaneous and second malignancy.

**S5.** Logistic regression model estimates (outcome = concordance between medical survey and LSQ answers).

|  |  |  | | |  | | |  | | |  | | | |
| --- | --- | --- | --- | --- | --- | --- | --- | --- | --- | --- | --- | --- | --- | --- |
|  | | **Sex**  (Ref: Male) | | | **Age at treatment start**  (Ref: 40 years and above) | | | **Clinical stage**  (Ref: Early stage) | | | **Educational level**  (Ref: University degree) | | | |
|  | |  | | |  | | |  | | |  | | | |
|  |  | OR | 95% CI | p-value | OR | 95% CI | p-value | OR | 95% CI | p-value | OR | 95% CI | | p-value |
|  | |  |  |  |  |  |  |  |  |  |  |  | |  |
| **Cardiovascular** | |  |  |  |  |  |  |  |  |  |  |  | |  |
|  | Myocardial infarction * | 1.60 | 0.95-2.70 | 0.079 | 2.89 | 1.72-4.84 | <0.001 | 0.99 | 0.47-2.06 | 0.970 | 0.35 | 0.17-0.69 | | 0.002 |
|  | Congestive heart failure * | 0.94 | 0.66-1.35 | 0.745 | 2.28 | 1.58-3.30 | <0.001 | 1.76 | 0.94-3.28 | 0.758 | 0.57 | 0.38-0.86 | | 0.006 |
|  | Rhythm abnormalities * | 1.14 | 0.77-1.68 | 0.513 | 1.52 | 1.00-2.31 | 0.049 | 0.74 | 0.44-1.26 | 0.266 | 0.75 | 0.49-1.15 | | 0.192 |
|  | Valvular disease * | 0.72 | 0.48-1.09 | 0.121 | 1.03 | 0.64-1.63 | 0.914 | 4.05 | 1.47-11.20 | 0.006 | 0.56 | 0.35-0.89 | | 0.014 |
|  | Hypertension * | 1.14 | 0.83-1.56 | 0.418 | 1.91 | 1.37-2.67 | <0.001 | 1.20 | 0.74-1.95 | 0.465 | 0.67 | 0.47-0.94 | | 0.022 |
|  | Stroke * | 0.77 | 0.41-1.44 | 0.409 | 2.48 | 1.31-4.70 | 0.005 | 1.39 | 0.49-3.98 | 0.539 | 0.46 | 0.21-1.03 | | 0.057 |
| **Pulmonary** | |  |  |  |  |  |  |  |  |  |  |  | |  |
|  | Pulmonary embolism * | 1.74 | 0.63-4.85 | 0.288 | 1.59 | 0.56-4.49 | 0.379 | 2.39 | 0.31-18.29 | 0.400 | 0.60 | 0.19-1.89 | | 0.379 |
|  | Pleuritis/pleural effusion * | 0.75 | 0.34-1.66 | 0.483 | 0.95 | 0.37-2.43 | 0.920 | 0.78 | 0.26-2.31 | 0.653 | 1.27 | 0.57-2.82 | | 0.554 |
|  | Pulmonary function altered * | 0.61 | 0.43-0.87 | 0.006 | 1.16 | 0.78-1.71 | 0.471 | 1.47 | 0.82-2-62 | 0.196 | 0.80 | 0.55-1.15 | | 0.229 |
| **Digestive tract** | |  |  |  |  |  |  |  |  |  |  |  | |  |
|  | Gastric antral stenosis | 1.28 | 0.77-2.13 | 0.342 | 1.36 | 0.78-2.37 | 0.277 | 2.36 | 0.84-6.60 | 0.102 | 1.06 | 0.62-1.80 | | 0.832 |
|  | Bowel (sub)obstruction | 0.43 | 0.13-1.41 | 0.164 | 1.65 | 0.49-5.57 | 0.417 | 0.72 | 0.16-3.30 | 0.669 | 2.23 | 0.73-6.85 | | 0.159 |
|  | Gastric or duodenal ulcer | 1.27 | 0.66-2.47 | 0.472 | 0.88 | 0.41-1.91 | 0.750 | 1.77 | 0.54-5.84 | 0.049 | 0.70 | 0.34-1.44 | | 0.327 |
|  | Bowel perforation * | 1.00 | 0.20-5.04 | 0.997 | 2.64 | 0.52-13.51 | 0.243 | 10.0 | 0.00-inf | 0.991 | 0.39 | 0.04-3.44 | | 0.397 |
| **Endocrine** | |  |  |  |  |  |  |  |  |  |  |  | |  |
|  | Hypothyroidism * | 0.66 | 0.49-0.87 | 0.003 | 0.94 | 0.67-1.31 | 0.716 | 2.69 | 1.55-4.69 | <0.001 | 1.07 | 0.79-1.43 | | 0.671 |
|  | Diabetes mellitus (post treatment) * | 2.72 | 1.50-4.93 | <0.001 | 4.13 | 2.39-7.14 | <0.001 | 1.22 | 0.53-2.78 | 0.642 | 0.63 | 0.33-1.17 | | 0.144 |
| **Urologic** | |  |  |  |  |  |  |  |  |  |  |  | |  |
|  | Renal insufficiency  (> doubling of s-creatinine) | 1.51 | 0.69-3.30 | 0.299 | 0.97 | 0.40-2.35 | 0.947 | 0.67 | 0.25-1.81 | 0.434 | 0.85 | 0.38-1.94 | | 0.707 |
| **Neurologic** | |  |  |  |  |  |  |  |  |  |  |  | |  |
|  | Peripheral polyneuropathy * | 0.77 | 0.50-1.20 | 0.244 | 2.37 | 1.51-3.74 | <0.001 | 0.77 | 0.42-1.40 | 0.391 | 1.17 | 0.74-1.85 | | 0.496 |
| **Musculoskeletal** | |  |  |  |  |  |  |  |  |  |  |  | |  |
|  | Avascular necrosis of the bone | 1.34 | 0.42-4.29 | 0.621 | 3.13 | 0.98-10.06 | 0.055 | 1.71 | 0.22-13.39 | 0.611 | 1.47 | 0.45-4.76 | | 0.524 |
|  | Musculo/(sub)cutaneous atrophy in irradiated areas * | 0.51 | 0.30-0.87 | 0.013 | 0.57 | 0.28-1.14 | 0.109 | 1.40 | 0.59-3.32 | 0.440 | 1.39 | 0.84-2.31 | | 0.195 |
| **Oral** | |  |  |  |  |  |  |  |  |  |  |  | |  |
|  | Xerostomia (>1 year) | 0.60 | 0.43-0.84 | 0.003 | 1.68 | 0.43-0.84 | 0.003 | 1.68 | 1.17-2.41 | 0.005 | 0.86 | 0.60-1.22 | | 0.391 |
|  | Dental sequelae | 0.91 | 0.59-1.41 | 0.681 | 0.98 | 0.59-1.63 | 0.681 | 0.72 | 0.40-1.28 | 0.261 | 1.26 | 0.81-1.96 | | 0.299 |
| **Miscellaneous** | |  |  |  |  |  |  |  |  |  |  |  | |  |
|  | Raynaud phenomenon (req. medication) | 0.41 | 0.16-1.02 | 0.055 | 2.36 | 0.97-5.71 | 0.057 | 0.28 | 0.11-0.71 | 0.007 | 1.48 | 0.61-3.58 | | 0.382 |
|  | Persistent fatigue * | 0.66 | 0.50-0.87 | 0.003 | 1.01 | 0.74-1.38 | 0.955 | 0.85 | 0.57-1.26 | 0.420 | 0.94 | 0.70-1.25 | | 0.658 |
|  | Depression/anxiety * | 0.74 | 0.53-1.03 | 0.074 | 0.80 | 0.53-1.19 | 0.272 | 1.31 | 0.77-2.24 | 0.316 | 1.14 | 0.81-1.60 | | 0.454 |
|  | Suicide attempt | 1.21 | 0.48-3.01 | 0.684 | 0.87 | 0.48-3.01 | 0.684 | 2.65 | 0.35-20.10 | 0.345 | 1.86 | 0.74-4.66 | | 0.187 |
| **Second malignancy** | |  |  |  |  |  |  |  |  |  |  |  | |  |
|  | Any second malignancy * | 0.97 | 0.68-1.38 | 0.869 | 1.02 | 0.68-1.53 | 0.930 | 1.12 | 0.65-1.93 | 0.678 | 0.83 | 0.57-1.21 | | 0.327 |
|  |  |  |  |  |  |  |  |  |  |  |  | |  | |

OR: odds ratio

CI: confidence interval

*Dates truncated in the medical survey to match the LSQ (dates not available for the conditions without an asterisk).

**S6**. Analyses of agreement (complete cases)

|  |  | |  | | | | | | | |  | | | | |
| --- | --- | --- | --- | --- | --- | --- | --- | --- | --- | --- | --- | --- | --- | --- | --- |
|  | | |  | | **2 x 2 Table** | | | | | |  | | | | |
|  | | |  | | | | | | | |  | | | | |
|  | | |  | | **Response in the Medical survey (dates truncated to match the LSQ)** | | | | | | **Response in the Medical survey (all available data)** | | | | |
|  | | |  | |  | | | |  | |  | | | | |
|  |  | | **Response in the LSQ** | | No | | Yes | p-value^ | *K* (95 % CI) | **Response in the LSQ** | No | | Yes | p-value^ | *K* (95 % CI) |
|  | | |  | |  | | | |  | |  | | | | |
| **Cardiovascular** | | |  | |  | | | |  | |  | | | | |
|  | | |  | |  | | | |  | |  | | | | |
|  | Myocardial infarction | | No | |  | 1124 | 15 | 0.027 | 0.55 (0.43-0.66) | No |  | 1124 | 23 | 0.3408 | 0.52 (0.41-0.63) |
|  |  | | Yes | |  | 31 | 30 |  | Yes | |  | 31 | 31 |  | |
|  |  | |  | |  | |  | |  | |  |  |  |  | |
|  | Congestive heart failure | | No | |  | 1043 | 12 | <0.001 | 0.22 (0.13-0.30) | No |  | 1043 | 25 | <0.001 | 0.23 (0.15-0.31) |
|  |  | | Yes | |  | 119 | 22 |  |  | Yes |  | 119 | 28 |  | |
|  |  | |  | |  |  |  | |  | |  |  |  |  | |
|  | Rhythm abnormalities | | No | |  | 1067 | 24 | <0.001 | 0.31 (0.21-0.40) | No |  | 1067 | 43 | <0.001 | 0.25 (0.16-0.33) |
|  |  | | Yes | |  | 91 | 28 |  |  | Yes |  | 91 | 29 |  | |
|  |  | |  | |  |  |  | |  | |  |  |  |  | |
|  | Valvular disease | | No | |  | 1036 | 42 | 0.051 | 0.25 (0.16-0.34) | No |  | 1037 | 78 | 0.2384 | 0.36 (0.28-0.44) |
|  |  | | Yes | |  | 63 | 48 |  |  | Yes |  | 63 | 52 |  | |
|  |  | |  | |  |  |  | |  | |  |  |  |  | |
|  | Hypertension | | No | |  | 977 | 47 | <0.001 | 0.25 (0.17-0.32) | No |  | 977 | 59 | <0.001 | 0.24 (0.17-0.31) |
|  |  | | Yes | |  | 134 | 43 |  |  | Yes |  | 134 | 46 |  | |
|  |  | |  | |  |  |  | |  | |  |  |  |  | |
|  | Stroke | | No | |  | 1170 | 5 | 0.001 | 0.35 (0.17-0.53) | No |  | 1170 | 11 | 0.04252 | 0.30 (0.13-0.47) |
|  |  | | Yes | |  | 24 | 8 |  |  | Yes |  | 24 | 8 |  | |
| **Pulmonary** | | |  | |  |  |  | |  | |  |  |  |  | |
|  | Pulmonary embolism | | No | |  | 1200 | 2 | 0.005 | 0.55 (0.35-0.75) | No |  | 1200 | 6 | 0.1175 | 0.49 (0.30-0.68) |
|  |  | | Yes | |  | 14 | 10 |  |  | Yes |  | 14 | 10 |  | |
|  |  | |  | |  |  |  | |  | |  |  |  |  | |
|  | Pleuritis/pleural effusion | | No | |  | 1195 | 4 | 0.001 | 0.23 (0.04-0.42) | No |  | 1196 | 8 | 0.01762 | 0.20 (0.03-0.37) |
|  |  | | Yes | |  | 22 | 4 |  |  | Yes |  | 22 | 4 |  | |
|  |  | |  | |  |  |  | |  | |  |  |  |  | |
|  | Pulmonary function altered | | No | |  | 1057 | 16 | <0.001 | 0.08 (0.02-0.15) | No |  | 1057 | 26 | <0.001 | 0.09 (0.02-0.15) |
|  |  | | Yes | |  | 135 | 10 |  |  | Yes |  | 135 | 12 |  | |
| **Digestive tract** | | |  | |  |  |  |  |  | |  |  |  |  | |
|  | Gastric antral stenosis | | No | |  | Not available | |  |  | No |  | 1163 | 1 | <0.001 | 0.03 (-0.03-0.08) |
|  |  | | Yes | |  |  |  |  |  | Yes |  | 65 | 1 |  | |
|  |  | |  | |  |  |  |  |  | |  |  |  |  | |
|  | Bowel (sub)obstruction | | No | |  | Not available | |  |  | No |  | 1216 | 2 | 0.0265 | 0.13 (-0.10-0.36) |
|  |  | | Yes | |  |  |  |  |  | Yes |  | 11 | 1 |  | |
|  |  | |  | |  |  |  |  |  | |  |  |  |  | |
|  | Gastric or duodenal ulcer | | No | |  | Not available | |  |  | No |  | 1192 | 7 | <0.001 | -0.01 (-0.02-[-0.00]) |
|  |  | | Yes | |  |  |  |  |  | Yes |  | 31 | 0 |  | |
|  |  | |  | |  |  |  |  |  | |  |  |  |  | |
|  | Bowel perforation | | No | |  | 1224 | 0 | <0.001 | 0.0 (-1.00 - 1.00) | No |  | 1224 | 0 | <0.001 | 0.0 (-1.00 - 1.00) |
|  |  | | Yes | |  | 6 | 0 |  |  | Yes |  | 6 | 0 |  | |
| **Endocrine** | | |  | |  |  |  |  |  | |  |  |  |  | |
|  | Hypothyroidism | | No | |  | 774 | 34 | <0.001 | 0.48 (0.43-0.54 | No |  | 774 | 53 | <0.001 | 0.46 (0.41-0.52) |
|  |  | | Yes | |  | 197 | 173 |  |  | Yes |  | 197 | 180 |  | |
|  |  | |  | |  |  |  |  |  | |  |  |  |  | |
|  | Diabetes mellitus | | No | |  | 774 | 34 | <0.001 | 0.51 (0.40-0.63) | No |  | 1135 | 14 | <0.001 | 0.48 (0.37-0.60) |
|  |  | | Yes | |  | 197 | 173 |  |  | Yes |  | 43 | 29 |  | |
| **Urologic** | | |  | |  |  |  |  |  | |  |  |  |  | |
|  | Renal insufficiency | | No | |  | Not available | |  |  | No |  | 1195 | 11 | 0.3447 | 0.32 (0.14-0.51) |
|  |  | | Yes | |  |  |  |  |  | Yes |  | 17 | 7 |  | |
| **Neurologic** | | |  | |  |  |  |  |  | |  |  |  |  | |
|  | Peripheral polyneuropathy | | No | |  | 1130 | 16 | <0.001 | 0.15 (0.05-0.25) | No |  | 1130 | 22 | <0.001 | 0.15 (0.05-0.25) |
|  |  | | Yes | |  | 68 | 9 |  |  | Yes |  | 68 | 10 |  | |
| **Musculoskeletal** | | |  | |  |  |  |  |  | |  |  |  |  | |
|  | Avascular necrosis of the bone | | No | |  | Not available | |  |  | No |  | 1215 | 1 | 0.0094 | 0.33 (0.05-0.61) |
|  |  | | Yes | |  |  |  |  |  | Yes |  | 11 | 3 |  | |
|  |  | |  | |  |  |  |  |  | |  |  |  |  | |
|  | Musculo/(sub)cutaneous atrophy… | | No | |  | 1149 | 10 | <0.001 | 0.09 (-0.01-0.18) | No |  | 1149 | 19 | <0.001 | 0.07 (-0.02-0.15) |
|  |  | | Yes | |  | 58 | 4 |  |  | Yes |  | 58 | 4 |  | |
| **Oral** | | |  | |  |  |  |  |  | |  |  |  |  | |
|  | Xerostomia (>1 year) | | No | |  | Not available | |  |  | No |  | 1056 | 1 | <0.001 | 0.03 (-0.01-0.06) |
|  |  | | Yes | |  |  |  |  |  | Yes |  | 170 | 3 |  | |
|  |  | |  | |  |  |  |  |  | |  |  |  |  | |
|  | Dental sequelae | | No | |  | Not available | |  |  | No |  | 1132 | 16 | <0.001 | 0.11 (0.019-0.20) |
|  |  | | Yes | |  |  |  |  |  | Yes |  | 75 | 7 |  | |
| **Miscellaneous** | | |  | |  |  |  |  |  | |  |  |  |  | |
|  | Raynaud phenomenon (req. medication) | | No | |  | Not available | |  |  | No |  | 1207 | 2 | 0.0002 | -0.00 (-0.00-[-0.00]) |
|  |  | | Yes | |  |  |  |  |  | Yes |  | 21 | 0 |  | |
|  |  | |  | |  |  |  |  |  | |  |  |  |  | |
|  | Persistent fatigue | | No | |  | 895 | 46 | <0.001 | 0.12 (0.07-0.18) | No |  | 895 | 58 | <0.001 | 0.13 (0.07-0.18) |
|  |  | | Yes | |  | 233 | 38 |  |  | Yes |  | 233 | 44 |  | |
|  |  | |  | |  |  |  |  |  | |  |  |  |  | |
|  | Depression/anxiety | | No | |  | 1030 | 27 | <0.001 | 0.13 (0.06-0-20) | No |  | 1030 | 35 | <0.001 | 0.15 (0.08-0.22) |
|  |  | | Yes | |  | 142 | 19 |  |  | Yes |  | 142 | 23 |  |  |
|  |  | |  | |  |  |  |  |  | |  |  |  |  | |
|  | Suicide attempt | | No | |  |  | |  |  | No |  | 1206 | 4 | 0.02178 | 0.34 (0.11-0.56) |
|  |  | | Yes | |  | Not available | |  |  | Yes |  | 15 | 5 |  |  |
| **Second malignancy** | | |  | |  |  |  |  |  |  |  |  |  |  | |
|  | Any second malignancy | | No | |  | 890 | 65 | 0.140 | 0.50 (0.43-0.57) | No |  | 893 | 140 | <0.001 | 0.39 (0.32-0.45) |
|  |  | | Yes | |  | 84 | 103 |  |  | Yes |  | 85 | 112 |  |  |
|  |  |  | |  |  | | | | | |  | | | | |

^ McNemar’s Chi-squared test with continuity correction

**S7**. Analyses of agreement stratified by sex (complete cases)

|  |  | |  | | | | | | | |  | | | | |
| --- | --- | --- | --- | --- | --- | --- | --- | --- | --- | --- | --- | --- | --- | --- | --- |
|  | | |  | | **2 x 2 Table** | | | | | |  | | | | |
|  | | |  | | | | | | | |  | | | | |
|  | | |  | | **Response in the Medical survey (Males)** | | | | | | **Response in the Medical survey (Females)** | | | | |
|  | | |  | |  | | | |  | |  | | | | |
|  |  | | **Response in the LSQ** | | No | | Yes | p-value^ | *K* (95 % CI) | **Response in the LSQ** | No | | Yes | p-value^ | *K* (95 % CI) |
|  | | |  | |  | | | |  | |  | | | | |
| **Cardiovascular** | | |  | |  | | | |  | |  | | | | |
|  | | |  | |  | | | |  | |  | | | | |
|  | Myocardial infarction | | No | |  | 529 | 17 | 0.742 | 0.57 (0.44-0.69) | No |  | 595 | 6 | 0.332 | 0.31 (0.07-0.55) |
|  |  | | Yes | |  | 20 | 28 |  | Yes | |  | 11 | 4 |  | |
|  |  | |  | |  | |  | |  | |  |  |  |  | |
|  | Congestive heart failure | | No | |  | 516 | 11 | <0.001 | 0.21 (0.09-0.33) | No |  | 527 | 14 | <0.001 | 0.25 (0.13-0.36) |
|  |  | | Yes | |  | 58 | 12 |  |  | Yes |  | 61 | 16 |  | |
|  |  | |  | |  |  |  | |  | |  |  |  |  | |
|  | Rhythm abnormalities | | No | |  | 518 | 24 | 0.007 | 0.20 (0.09-0.32) | No |  | 549 | 19 | 0.003 | 0.29 (0.16-0.42) |
|  |  | | Yes | |  | 48 | 13 |  |  | Yes |  | 43 | 16 |  | |
|  |  | |  | |  |  |  | |  | |  |  |  |  | |
|  | Valvular disease | | No | |  | 523 | 29 | 0.787 | 0.43 (0.30-0.55) | No |  | 514 | 49 | 0.236 | 0.31 (0.20-0.42) |
|  |  | | Yes | |  | 26 | 25 |  |  | Yes |  | 37 | 27 |  | |
|  |  | |  | |  |  |  | |  | |  |  |  |  | |
|  | Hypertension | | No | |  | 477 | 27 | <0.001 | 0.23 (0.13-0.34) | No |  | 500 | 32 | 0.001 | 0.25 (0.14-0-35) |
|  |  | | Yes | |  | 69 | 22 |  |  | Yes |  | 65 | 24 |  | |
|  |  | |  | |  |  |  | |  | |  |  |  |  | |
|  | Stroke | | No | |  | 576 | 7 | 0.628 | 0.31 (0.07-0.55) | No |  | 594 | 4 | 0.034 | 0.30 (0.06-0.53) |
|  |  | | Yes | |  | 10 | 4 |  |  | Yes |  | 14 | 4 |  | |
| **Pulmonary** | | |  | |  |  |  | |  | |  |  |  |  | |
|  | Pulmonary embolism | | No | |  | 585 | 4 | 0.387 | 0.49 (0.24-0.75) | No |  | 615 | 2 | 0.289 | 0.49 (0.19-0.80) |
|  |  | | Yes | |  | 8 | 6 |  |  | Yes |  | 6 | 4 |  | |
|  |  | |  | |  |  |  | |  | |  |  |  |  | |
|  | Pleuritis/pleural effusion | | No | |  | 586 | 6 | 0.789 | 0.29 (0.02-0.55) | No |  | 610 | 2 | 0.006 | 0.1 (-0.09-0.30) |
|  |  | | Yes | |  | 8 | 3 |  |  | Yes |  | 14 | 1 |  | |
|  |  | |  | |  |  |  | |  | |  |  |  |  | |
|  | Pulmonary function altered | | No | |  | 535 | 15 | <0.001 | 0.07 (-0.03-0.17) | No |  | 522 | 11 | <0.001 | 0.09 (0.02-0.18) |
|  |  | | Yes | |  | 49 | 4 |  |  | Yes |  | 86 | 8 |  | |
| **Digestive tract** | | |  | |  |  |  |  |  | |  |  |  |  | |
|  | Gastric antral stenosis | | No | |  | 567 | 0 | <0.001 | 0.05 (-0.05-0.15) | No |  | 596 | 1 | <0.001 | 0.00 (-0.01-0.01) |
|  |  | | Yes | |  | 35 | 1 |  |  | Yes |  | 30 | 0 |  | |
|  |  | |  | |  |  |  |  |  | |  |  |  |  | |
|  | Bowel (sub)obstruction | | No | |  | 599 | 1 | 0.617 | -0.00 (-0.01-0.00) | No |  | 617 | 1 | 0.046 | 0.18 (-0.13-0.48) |
|  |  | | Yes | |  | 3 | 0 |  |  | Yes |  | 8 | 1 |  | |
|  |  | |  | |  |  |  |  |  | |  |  |  |  | |
|  | Gastric or duodenal ulcer | | No | |  | 583 | 3 | 0.004 | -0.01 (-0.02-0.00) | No |  | 609 | 4 | 0.034 | -0.01 (-0.02-0.00) |
|  |  | | Yes | |  | 17 | 0 |  |  | Yes |  | 14 | 0 |  | |
|  |  | |  | |  |  |  |  |  | |  |  |  |  | |
|  | Bowel perforation | | No | |  | 600 | 0 | 0.034 | 0.0 (-1.00-1.00) | No |  | 624 | 0 | 0.034 | 0.00 (0.00-0.00) |
|  |  | | Yes | |  | 3 | 0 |  |  | Yes |  | 3 | 0 |  | |
| **Endocrine** | | |  | |  |  |  |  |  | |  |  |  |  | |
|  | Hypothyroidism | | No | |  | 424 | 22 | <0.001 | 0.49 (0.41-0.58) | No |  | 350 | 31 | <0.001 | 0.43 (0.35-0.50) |
|  |  | | Yes | |  | 74 | 69 |  |  | Yes |  | 123 | 111 |  | |
|  |  | |  | |  |  |  |  |  | |  |  |  |  | |
|  | Diabetes mellitus | | No | |  | 536 | 11 | 0.003 | 0.45 (0.31-0.59) | No |  | 599 | 3 | 0.039 | 0.53 (0.33-0.74) |
|  |  | | Yes | |  | 31 | 20 |  |  | Yes |  | 12 | 9 |  | |
| **Urologic** | | |  | |  |  |  |  |  | |  |  |  |  | |
|  | Renal insufficiency | | No | |  | 583 | 7 | 0.803 | 0.32 (0.07-0.57) | No |  | 612 | 4 | 0.387 | 0.32 (0.04-0.61) |
|  |  | | Yes | |  | 9 | 4 |  |  | Yes |  | 8 | 3 |  | |
| **Neurologic** | | |  | |  |  |  |  |  | |  |  |  |  | |
|  | Peripheral polyneuropathy | | No | |  | 556 | 14 | 0.082 | 0.23 (0.07-0.38) | No |  | 574 | 8 | <0.001 | 0.08 (-0.03-0.19) |
|  |  | | Yes | |  | 26 | 7 |  |  | Yes |  | 42 | 3 |  | |
| **Musculoskeletal** | | |  | |  |  |  |  |  | |  |  |  |  | |
|  | Avascular necrosis of the bone | | No | |  | 594 | 1 | 0.131 | 0.36 (-0.00-0.72) | No |  | 621 | 0 | 0.074 | 0.28 (-0.15-0.72) |
|  |  | | Yes | |  | 6 | 2 |  |  | Yes |  | 5 | 1 |  | |
|  |  | |  | |  |  |  |  |  | |  |  |  |  | |
|  | Musculo/(sub)cutaneous atrophy… | | No | |  | 575 | 8 | 0.078 | 0.11 (-0.05-0.28) | No |  | 574 | 11 | <0.001 | 0.04 (-0.05-0.14) |
|  |  | | Yes | |  | 18 | 2 |  |  | Yes |  | 40 | 2 |  | |
| **Oral** | | |  | |  |  |  |  |  | |  |  |  |  | |
|  | Xerostomia (>1 year) | | No | |  | 534 | 0 | <0.001 | 0.05 (-0.02-0.12) | No |  | 522 | 1 | <0.001 | 0.01 (-0.02-0.04) |
|  |  | | Yes | |  | 67 | 2 |  |  | Yes |  | 103 | 1 |  | |
|  |  | |  | |  |  |  |  |  | |  |  |  |  | |
|  | Dental sequelae | | No | |  | 557 | 9 | <0.001 | 0.09 (-0.03-0.22) | No |  | 575 | 7 | <0.001 | 0.12 (-0.00-0.24) |
|  |  | | Yes | |  | 34 | 3 |  |  | Yes |  | 41 | 4 |  | |
| **Miscellaneous** | | |  | |  |  |  |  |  | |  |  |  |  | |
|  | Raynaud phenomenon (req. medication) | | No | |  | 596 | 0 | <0.001 | 0.00 (0.00-0.00) | No |  | 611 | 2 | 0.006 | -0.00 (-0.01-0.001) |
|  |  | | Yes | |  | 7 | 0 |  |  | Yes |  | 14 | 0 |  | |
|  |  | |  | |  |  |  |  |  | |  |  |  |  | |
|  | Persistent fatigue | | No | |  | 471 | 24 | <0.001 | 0.07 (-0-00-0.15) | No |  | 424 | 34 | <0.001 | 0.15 (0.07-0.23) |
|  |  | | Yes | |  | 97 | 11 |  |  | Yes |  | 136 | 33 |  | |
|  |  | |  | |  |  |  |  |  | |  |  |  |  | |
|  | Depression/anxiety | | No | |  | 524 | 15 | <0.001 | 0.07 (-0.02-0.17) | No |  | 506 | 20 | <0.001 | 0.19 (0.09-0.28) |
|  |  | | Yes | |  | 59 | 5 |  |  | Yes |  | 83 | 18 |  |  |
|  |  | |  | |  |  |  |  |  | |  |  |  |  | |
|  | Suicide attempt | | No | |  | 589 | 3 | 0.343 | 0.44 (0.15-0.72) | No |  | 617 | 1 | 0.046 | 0.18 (-0.13-0.48) |
|  |  | | Yes | |  | 7 | 4 |  |  | Yes |  | 8 | 1 |  |  |
| **Second malignancy** | | |  | |  |  |  |  |  |  |  |  |  |  | |
|  | Any second malignancy | | No | |  | 461 | 59 | 0.111 | 0.35 (0.25-0.45) | No |  | 432 | 81 | 0.001 | 0.41 (0.33-0.50) |
|  |  | | Yes | |  | 42 | 41 |  |  | Yes |  | 43 | 71 |  |  |
|  |  |  | |  |  | | | | | |  | | | | |

^ McNemar’s Chi-squared test with continuity correction.

**S8.** Cohens Kappa coefficients (rate of agreement) according to subgroup.

|  | |  |  |  |  |  |  |
| --- | --- | --- | --- | --- | --- | --- | --- |
|  | | **Kappa coefficients (95% CI)** | | | | |  |
|  | |  | | | |  |  |
|  | | Total cohort | Subgroup  (males) | Subgroup  (females) | Subgroup  (dates truncated) | Subgroup  (requiring medication) | Subgroup  (dates truncated + req. medication) |
|  | |  |  |  |  |  |  |
|  | |  |  |  |  |  |  |
|  | |  |  |  |  |  |  |
| **Cardiovascular** | |  |  |  |  |  |  |
|  | Myocardial infarction | 0.52 (0.41-0.63) | 0.57 (0.44-0.69) | 0.31 (0.07-0.55) | 0.55 (0.43-0.66) | - | - |
|  | Congestive heart failure | 0.23 (0.15-0.31) | 0.21 (0.09-0.33) | 0.25 (0.13-0.36) | 0.22 (0.13-0.30) | 0.16 (0.08-0.24) | 0.14 (0.06-0.22) |
|  | Rhythm abnormalities | 0.25 (0.16-0.33) | 0.20 (0.09-0.32) | 0.29 (0.16-0.42) | 0.31 (0.21-0.40) | 0.30 (0.19-0.41) | 0.32 (0.21-0.43) |
|  | Valvular disease | 0.36 (0.28-0.44) | 0.43 (0.30-0.55) | 0.31 (0.20-0.42) | 0.25 (0.16-0.34) | 0.26 (0.15-0.38) | 0.28 (0.16-0.40) |
|  | Hypertension | 0.24 (0.17-0.31) | 0.23 (0.13-0.34) | 0.25 (0.14-0-35) | 0.25 (0.17-0.32) | 0.17 (0.10-0.24) | 0.18 (0.11-0.25) |
|  | Stroke | 0.30 (0.13-0.47) | 0.31 (0.07-0.55) | 0.30 (0.06-0.53) | 0.35 (0.17-0.53) | 0.12 (-0.04-0.29) | 0.15 (-0.04-0.34) |
| **Pulmonary** | |  |  |  |  |  |  |
|  | Pulmonary embolism | 0.49 (0.30-0.68) | 0.49 (0.24-0.75) | 0.49 (0.19-0.80) | 0.55 (0.35-0.75) | 0.46 (0.26-0.67) | 0.51 (0.30-0.73) |
|  | Pleuritis/pleural effusion | 0.20 (0.03-0.37) | 0.29 (0.02-0.55) | 0.10 (-0.09-0.30) | 0.23 (0.04-0.42) | 0.06 (-0.07-0.18) | 0.07 (-0.07-0.20) |
|  | Pulmonary function altered | 0.09 (0.02-0.15) | 0.07 (-0.03-0.17) | 0.09 (0.02-0.18) | 0.08 (0.02-0.15) | 0.05 (-0.01-0.12) | 0.04 (-0.02-0.09) |
| **Digestive tract** | |  |  |  |  |  |  |
|  | Gastric antral stenosis | 0.03 (-0.03-0.08) | 0.05 (-0.05-0.15) | 0.00 (-0.01-0.01) | - | 0.04 (-0.04-0.13) | - |
|  | Bowel (sub)obstruction | 0.13 (-0.10-0.36) | -0.00 (-0.01-0.00) | 0.18 (-0.13-0.48) | - | -0.001 (-0.004-0.0011) | - |
|  | Gastric or duodenal ulcer | -0.01 (-0.02-0.00]) | -0.01 (-0.02-0.00) | -0.01 (-0.02-0.00) | - | -0.006 (-0.011-[-1.00]) | - |
|  | Bowel perforation | 0.0 (-1.00-1.00) | 0.00 (-1.00-1.00) | 0.00 (0.00-0.00) | 0.0 (-1.00 - 1.00) | 0.0 (-1.00-1.00) | 0.0 (-1.00-1.00) |
| **Endocrine** | |  |  |  |  |  |  |
|  | Hypothyroidism | 0.46 (0.41-0.52) | 0.49 (0.41-0.58) | 0.43 (0.35-0.50) | 0.48 (0.43-0.54) | - | - |
|  | Diabetes mellitus (post treatment) | 0.48 (0.37-0.60) | 0.45 (0.31-0.59) | 0.53 (0.33-0.74) | 0.51 (0.40-0.63) | - | - |
| **Urologic** | |  |  |  |  |  |  |
|  | Renal insufficiency  (> doubling of s-creatinine) | 0.32 (0.14-0.51) | 0.32 (0.07-0.57) | 0.32 (0.04-0.61) | - | - | - |
| **Neurologic** | |  |  |  |  |  |  |
|  | Peripheral polyneuropathy | 0.15 (0.05-0.25) | 0.23 (0.07-0.38) | 0.08 (-0.03-0.19) | 0.15 (0.05-0.25) | - | - |
| **Musculoskeletal** | |  |  |  |  |  |  |
|  | Avascular necrosis of bone | 0.33 (0.05-0.61) | 0.36 (-0.00-0.72) | 0.28 (-0.15-0.72) | - | - | - |
|  | Musculo/(sub)cutaneous atrophy in irradiated areas | 0.07 (-0.02-0.15) | 0.11 (-0.05-0.28) | 0.04 (-0.05-0.14) | 0.09 (-0.01-0.18) | - | - |
| **Oral** | |  |  |  |  |  |  |
|  | Xerostomia (>1 year) | 0.03 (-0.01-0.06) | 0.05 (-0.02-0.12) | 0.01 (-0.02-0.04) | - | - | - |
|  | Dental sequelae | 0.11 (0.019-0.20) | 0.09 (-0.03-0.22) | 0.12 (-0.00-0.24) | - | - | - |
| **Miscellaneous** | |  |  |  |  |  |  |
|  | Raynaud phenomenon (req. medication) | -0.003 (-0.006-[-0.0008]) | 0.00 (0.00-0.00) | -0.00 (-0.01-0.001) | - | - | - |
|  | Persistent fatigue | 0.13 (0.07-0.18) | 0.07 (-0-00-0.15) | 0.15 (0.07-0.23) | 0.12 (0.07-0.18) | - | - |
|  | Depression/anxiety | 0.15 (0.08-0.22) | 0.07 (-0.02-0.17) | 0.19 (0.09-0.28) | 0.13 (0.06-0-20) | - | - |
|  | Suicide attempt | 0.34 (0.11-0.56) | 0.44 (0.15-0.72) | 0.18 (-0.13-0.48) | - | - | - |
| **Second malignancy** | |  |  |  |  |  |  |
|  | Any second malignancy | 0.39 (0.32-0.45) | 0.35 (0.25-0.45) | 0.41 (0.33-0.50) | 0.50 (0.43-0.57) | - | - |
|  | |  |  |  |  |  |  |
